# Effect of Exercise and Pulmonary Rehabilitation in Pre- and Post-Surgical Patients with Lung Cancer: Systematic Review and Meta-Analysis

**DOI:** 10.3390/medicina60111725

**Published:** 2024-10-22

**Authors:** Freiser Eceomo Cruz Mosquera, Saray Rios Murillo, Anisbed Naranjo Rojas, Claudia Lorena Perlaza, Diana Castro Osorio, Yamil Liscano

**Affiliations:** Grupo de Investigación en Salud Integral (GISI), Department of Health Sciences Faculty, Universidad Santiago de Cali, Cali 760035, Colombia; sarayrios00@usc.edu.co (S.R.M.); anisbed.naranjo00@usc.edu.co (A.N.R.); lorena.perlaza00@usc.edu.co (C.L.P.); d-marce36@hotmail.com (D.C.O.); yamil.liscano00@usc.edu.co (Y.L.)

**Keywords:** lung cancer, postoperative, exercise intervention, rehabilitation, non-small-cell lung cancer, length of stay, quality of life, mortality

## Abstract

*Background and objectives*: Lung cancer is a common cancer, and its impact on public health is not only reflected in the 1 million deaths it causes annually but also in the significant implications it has on daily activities and quality of life, resulting in a considerable burden on healthcare systems. This review aims to determine the effects of pulmonary rehabilitation and pre- or post-surgical exercise in patients with lung cancer. *Materials and methods*: A systematic review with a meta-analysis of randomized controlled trials published between 2010 and 2024 was conducted; the search was carried out in PubMed, Cochrane Clinical Trial, SCOPUS, Science Direct, Web of Science, Scielo, and LILAC. *Results*: Pulmonary rehabilitation or exercise before surgery was associated with a greater 6 min walking distance (MD: 37.42, 95% CI: 9.68–65.1; *p* = 0.008); however, it had no implications on hospital stay (MD: −0.91, 95% CI: −1.88–0.055; *p* = 0.06). When the intervention was performed post-surgery, higher FEV1 (SMD: 0.62, 95% CI: 0.32–0.92; *p* = 0.0001) and improved 6 min walking distances (60.8, 95% CI: 20.96–100.6; *p* = 0.0033) were found compared to standard management. *Conclusions*: This review suggests that, depending on the timing of implementation, pulmonary rehabilitation or exercise could produce positive effects on certain clinical variables in lung cancer patients.

## 1. Introduction

Lung cancer is defined as the abnormal growth of lung cells forming tumors in this large organ [1,2,3,4,5]. It is the most frequently diagnosed cancer worldwide, with an estimated mortality rate of up to 1.4 million cases per year; additionally, according to some studies, it is projected to reach 3 million deaths by 2035 [6]. Similarly, the International Agency for Research on Cancer (IARC) of the World Health Organization states that lung cancer is the most prevalent cancer globally, producing 2.5 million new cases and being the leading cause of cancer-related death, particularly among men [7,8]. Various studies indicate that the age-standardized lifetime risk of LC diagnosis is 3.8% for men and 1.77% for women [9,10].

Lung cancer has a higher incidence in developing countries where habits such as smoking are more prevalent, with significant variation between regions [7]. In countries like China, the incidence and mortality of lung cancer have increased, with 820,000 new cases and approximately 715,000 deaths reported since 2015 [11]. In contrast, in the United States, although this disease remains the most lethal type of cancer, both incidence and mortality have shown a decrease over the past two decades. Despite this, the disease burden remains significant [12]. In Latin America, in countries like Colombia, lung cancer is the sixth most frequent type, although the current trend suggests a slight reduction in rates. Annually, more than 6500 new cases of lung cancer are reported, with occupational and environmental exposure being the associated risk factors [13].

Based on the cells of origin, lung neoplasms can be divided into small cell lung cancer (SCLC) and non-small-cell lung cancer (NSCLC), the latter of which is further divided into several subtypes [14,15,16]. SCLC is typically an aggressive, poorly differentiated, high-grade neuroendocrine carcinoma that accounts for 13% of all lung carcinomas [17,18]. On the other hand, NSCLC is a heterogeneous group of tumors representing about 85% of all new lung cancer diagnoses; it is characterized by the fact that a large proportion of patients are diagnosed at advanced stages, among other reasons, due to the late onset of clinical manifestations, leading to a poor prognosis in most cases [19].

The impact of this condition is of public health interest, as it generates a substantial burden on the healthcare system, carries economic implications, and affects various dimensions of human life, leading to direct repercussions on family dynamics, the development of daily activities, and thus the quality of life of patients [20,21,22,23,24]. Traditionally, the cornerstone for improving the prognosis and quality of life of lung cancer patients has consisted of various interventions, including the elimination of harmful habits such as smoking, early detection of the disease, radiotherapy, chemotherapy, and surgery [25,26,27,28,29,30].

Despite advances in the treatment of lung cancer, a large proportion of patients are still diagnosed at advanced stages, which limits therapeutic options and negatively affects prognosis. In addition, traditional interventions, such as surgery and chemotherapy, are often not sufficient to significantly improve the quality of life of patients. In view of the foregoing, several authors have proposed that adding exercise to the strategies already described may have a positive impact on this population group. In this sense, in recent years, pre- and post-surgical pulmonary rehabilitation (PR) has gained importance [31]. PR is a comprehensive intervention based on evaluation and individualized therapies according to the requirements of each patient, including, but not limited to, physical training, breathing exercises, education, and lifestyle modification to positively impact the physical and psychological states of patients with lung diseases [32]. In lung cancer patients, PR is usually conducted in several phases: prehabilitation, perioperative rehabilitation, postoperative rehabilitation, and the maintenance phase [33]. In the preoperative phase, exercise and PR have been associated with numerous benefits, including increased functional capacity and exercise tolerance, thereby reducing fatigue and dyspnea and improving quality of life [34,35]. On the other hand, in the immediate postoperative period, it plays a fundamental role in the prevention of pulmonary complications that may arise after surgical intervention, thereby influencing morbidity, hospital stay, and medical costs [36,37].

This systematic review and meta-analysis aims to determine the effect of pulmonary rehabilitation and pre- or post-surgical exercise in lung cancer patients.

## 2. Materials and Methods

### 2.1. Study Protocol

The systematic review was conducted following the PRISMA statement guidelines for systematic reviews and meta-analyses [38,39] and the recommendations of the Cochrane Collaboration Handbook [40]. The research was formulated considering elements of the PICO strategy (Population, Intervention, Comparison, and Outcomes) [41].

### 2.2. Research Question

In patients with lung cancer (P), what are the effects of pulmonary rehabilitation and exercise (I) compared to standard treatment on quality of life, lung function, distance walked, symptom improvement, hospital stay, mortality, and adverse effects (O)?

### 2.3. Eligibility Criteria

#### 2.3.1. Inclusion Criteria

The inclusion criteria were as follows:

- Randomized controlled trials (RCTs);
- Studies published in Spanish and English;
- Studies published between January 2010 and March 2024;
- Adult patients diagnosed with lung cancer participating in pulmonary rehabilitation programs or undergoing exercise training of any intensity in any setting (hospital, community center, or home) before or after surgical procedures.
- Studies reporting at least one of the following outcomes: health-related quality of life, lung function, 6 min walking distance, symptom improvement, postoperative hospital stay, mortality, and adverse effects.

#### 2.3.2. Exclusion Criteria

The exclusion criteria were as follows:

- Preprint articles and letters to the editor;
- Studies published as conference abstracts;
- Studies not available in accessible formats;
- Patients with metastatic tumors or neoplasms;
- Patients with inoperable lung cancer due to advanced stage;
- Studies reporting the same patient cohort as previously published similar research.

### 2.4. Data Sources and Search Strategy

The search was conducted in the following databases and search engines: PubMed, Cochrane Clinical Trial, SCOPUS, Science Direct, Web of Science, Scielo, and LILAC. Filters were applied when necessary for Spanish and English languages and clinical trials, and the date range was set between 2010 and 2024. The search strategy was designed and executed from January to March 2024 by two independent researchers (SMR, YL) using the following keywords: Pulmonary Rehabilitation, Lung Training, Respiratory Rehabilitation, Lung Cancer, Lung Carcinoma, Lung Neoplasia, Effectiveness, Outcomes, and Benefits. The terms were combined using Boolean operators AND and OR. The search strategy is described as follows: (“Lung Rehabilitation” OR “Lung Training” OR “Respiratory Rehabilitation” OR “lung therapy” OR “rehabilitation program” OR “exercise”) AND (“Lung Cancer” OR “Lung Carcinoma” OR “Lung Neoplasia” OR “Lung Tumor”) AND (“Effectiveness” OR “Results” OR “Benefits” OR “Improvement”).

The references of relevant articles were reviewed, and additional web searches were conducted to identify studies not captured by the initial search. When necessary to confirm critical trial data, ClinicalTrials.gov (https://clinicaltrials.gov/, accessed 1 to 15 May 2024) was accessed for verification. Zotero version 6.0 was used for data storage.

During this process, any discrepancies among the authors were resolved through consensus achieved via discussions and, if necessary, with the intervention of a third reviewer to ensure objectivity and integrity in the subsequent selection of studies.

### 2.5. Study Selection and Data Extraction

The selection of potentially eligible studies was carried out independently by two researchers (DCO, CLP), initially reviewing the titles and abstracts, followed by the full texts. Studies with unclear relevance were discussed, and the decision to include them was made by consensus. To measure the degree of agreement among the authors and evaluate the consistency in the selection of studies, Cohen’s Kappa index was used [42]. The two reviewers extracted information from the primary studies, considering data detailing the research (first author, year of publication), participant characteristics (number of subjects, percentage of male patients, age), rehabilitation characteristics (timing of rehabilitation, program duration, activities performed, number of sessions, training setting), and outcomes (quality of life, lung function, 6 min walking distance, symptom improvement, hospital stay, mortality, and adverse events). Subsequently, three reviewers (FCM, YL, and ANR) verified the accuracy of the recorded information.

### 2.6. Risk of Bias Assessment

The risk of bias in the studies was independently assessed using a standardized tool that considers the essential elements of a clinical trial design. For the evaluation, data were recorded in Review Manager version 5.4^®^ (RevMan accessed on 15 to 23 May 2024). The criteria considered were (a) random sequence generation, (b) allocation concealment, (c) blinding of participants and personnel, (d) blinding of outcome assessment, (e) incomplete outcome data, and (f) selective reporting. For each domain evaluated, the RCT was considered to have a low, unclear, or high risk of bias, according to the adherence to predetermined guidelines [43]. Discrepancies in the risk of bias assessment were resolved through discussions among the reviewers until an agreement was reached.

### 2.7. Assessment of Evidence Quality

The quality of the trials included in the meta-analysis was evaluated by the reviewers using the Jadad scale [44]. The Jadad scale assigns each article a total score ranging from a minimum of 0 to a maximum of 5 (higher quality trials) based on the following criteria: (a) the study is randomized, (b) the intervention is double-blind, (c) withdrawals from the study are considered and described, (d) the randomization procedure is properly carried out using an appropriate method, such as computer-generated random numbers, and (e) the inclusion and exclusion criteria are clearly described. For each aspect, a score of 0 was given if it was not met or insufficiently described, and 1 if it was appropriately described in the methodology. A score equal to or greater than three was considered the cutoff point to define adequate quality of the clinical trials [45]. Studies were not excluded based on this evaluation, but their quality scores were reported as part of the results.

### 2.8. Statistical Analysis

Meta-analysis was performed by calculating the effect size, accompanied by the 95% confidence interval, using RevMan 5.4^®^ version software (RevMan accessed on 20 to 30 May 2024). Meta-analysis was conducted when there were three or more publications evaluating at least one of the mentioned outcomes. For reporting the findings, studies were separated based on whether the intervention was conducted before or after surgery. The outcomes included in the analysis were as follows: lung function determined by forced expiratory volume in one second (FEV1) and forced vital capacity (FVC) expressed in liters, 6 min walking distance measured in meters, and hospital stay after surgical intervention, particularly when rehabilitation was conducted before surgery. For each outcome, the mean and standard deviation were extracted from the primary study, or the respective transformation was performed if the report used the median and interquartile range. In cases where the outcome was measured at different time points, the last measurement was selected for both the intervention and control groups.

For all outcomes included in the meta-analysis, the mean difference (MD) or standardized mean difference (SMD) was used when trials measured the same outcome but applied different methods or units. Outcomes not included in the meta-analysis due to variability in reporting (quality of life, symptom improvement, adverse events, and mortality) were described qualitatively. Statistical heterogeneity was estimated using the I^2^ statistic. High heterogeneity was defined as I^2^ equal to or greater than 50%. Fixed and random effect models were used when I^2^ was less than or greater than 50%, respectively. Finally, a *p*-value < 0.05 was considered statistically significant. In the present study, a sensitivity analysis was not conducted.

## 3. Results

### 3.1. Studies Identified for the Review

**Figure 1:**
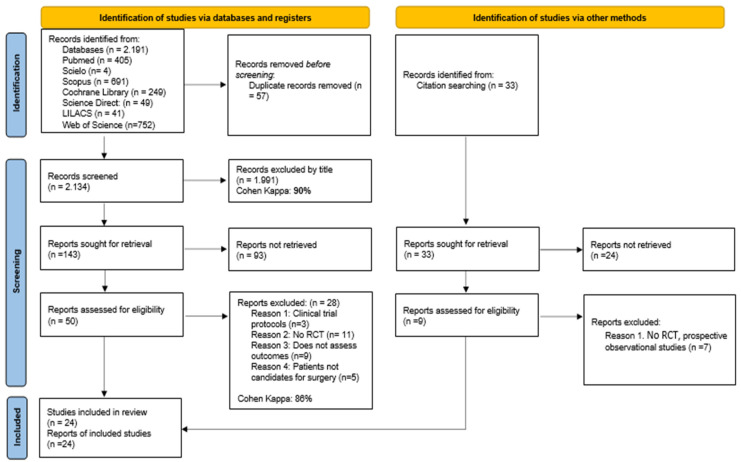
PRISMA flow diagram with the search and study selection strategy.

After conducting the search in the seven databases considered for the review, a total of 2191 potentially relevant articles were identified. Of these, 57 articles were removed as duplicates, leaving 2134 articles as the starting point. After excluding 1991 texts based on the title and 93 based on the abstract, a total of 50 articles were considered for full-text reading. Of the aforementioned research studies, 27 were excluded for the following reasons: (a) randomized controlled trial protocols (*n* = 3), (b) not randomized controlled trials (*n* = 10), (c) did not evaluate the proposed outcomes for this systematic review (*n* = 9), (d) patients were not candidates for surgical intervention due to their condition (*n* = 5). Finally, twenty-four articles were included in this review, two of which were identified through manual search. The details of the study selection process are provided in the PRISMA flow diagram (see Figure 1).

### 3.2. Characteristics of the Studies Included in the Review

All studies included in the review were randomized controlled trials; of these, 10 evaluated the effects of exercise-based rehabilitation before the surgical procedure [46,47,48,49,50,51,52,53,54,55] and 14 after surgery for lung cancer [56,57,58,59,60,61,62,63,64,65,66,67,68,69]. Most of the included clinical trials (*n* = 12/24) were published between 2020 and 2024, eight studies were published from 2015 to 2019, and four studies during the period from 2010 to 2014. Regarding the continent of publication, 50% of the scientific articles were reported in Europe, 38% in Asia, and the rest in America and Oceania. The three countries with the highest contribution of publications in this review were China (*n* = 7 studies) [46,48,50,56,57,60,61], Switzerland (*n* = 3 studies) [49,51,54], and Spain (*n* = 2 studies) [53,63].

The 10 clinical trials that evaluated the effects of pre-surgical rehabilitation or exercise included a total of 974 patients, of which 49% received the intervention proposed by the authors. The percentage of men in this population ranged from 34% to 90%, and the average age was 65.02 ± 4.54 years. Regarding the type of lung cancer evaluated, 90% of the studies included patients diagnosed with NSCLC, and 10% included subjects with NSCLC or SCLC. Moreover, the duration of the intervention varied slightly among the studies that reported it. Lai described the duration as 1 week of training. Lit et al. [46] and Bathia et al. [49] reported that patient engagement ranged from 2 to 3 weeks, while Morano et al. [52] reported 4 weeks of intervention. The remaining six studies did not clearly describe the duration of the intervention [47,48,51,53,54,55]. Regarding the evaluated outcomes, four studies examined lung function [46,50,52,54], seven studies evaluated the 6 min walking distance [46,48,49,50,51,52,55], five studies assessed hospital stay [46,50,51,52,55], three studies assessed symptom improvement [47,48,55], three studies evaluated adverse events associated with exercise [47,50,55], and one study assessed mortality [51]. Health-related quality of life was evaluated by five authors [46,47,50,53,55] using different instruments such as the Functional Assessment of Cancer Therapy—Lung (FACT-L), Quality of Life Questionnaire—Core 30 (QLQ-C30), Short Form 36 Health Survey (SF-36), and SF-12. Various methods of outcome reporting were identified. The synthesis of the mentioned studies is detailed in Table 1.

On the other hand, the 14 RCTs that examined the effects of exercise-based rehabilitation after surgery enrolled a total of 1094 patients, of which 547 (50%) were part of the intervention group. The percentage of male subjects averaged 58 ± 18.5%, and the average age was 63.8 ± 4.1 years. Regarding the pathologies of the studied patients, 71% of the studies included patients diagnosed with NSCLC, 21% included patients with NSCLC or SCLC, and the remaining studies included patients with simultaneous diagnoses of NSCLC and chronic obstructive pulmonary disease (COPD). Concerning the duration of exercise training, only one study did not clearly report it [56]. Ferreira et al. [58], Zhou et al. [59], Zou et al. [60], Liu et al. [61], Messaggi et al. [63], Rutkowsk et al. [64], Brocki et al. [65], Liu F et al. [62], and Granger et al. [69] reported a longer duration. Regarding the evaluated outcomes, 11 studies analyzed the 6 min walking distance [57]. Edvardsen et al. [66] and Stigt et al. [68] reported a longer duration. Regarding the evaluated outcomes, eleven studies analyzed the 6 min walking distance [56,57,58,59,60,61,62,64,65,68,69], eight studies evaluated lung function, seven studies reported health-related quality of life [56,58,63,66,67,68,69], four studies assessed hospital stay [58,59,60,61], three studies evaluated symptom improvement [59,60,64], and two studies determined mortality and adverse events, respectively [61,69]. See details in Table 2.

### 3.3. Summary of Intervention and Results of Studies Included in the Review

Table 3 describes the general characteristics of exercise-based rehabilitation from the studies where the intervention was conducted pre-surgery. It highlights the setting in which the intervention was implemented (medical center, home, or both), the number of sessions, and the activities performed during patient follow-up.

Of the 10 studies [46,47,48,49,50,51,52,53,54,55], in 70% of cases, the exercises were performed in a medical center under the strict supervision of healthcare professionals. In 20% of the studies, participants received the intervention both in the medical center and in the home setting, while in the remaining 10%, patients were specifically treated at home. Additionally, five studies did not clearly describe the number of sessions the patients underwent during the follow-up period [46,48,51,53,54]. In the remaining five studies, the reported number of sessions varied considerably, ranging from seven to as many as thirty-nine sessions [47,49,50,52,55]. Regarding the activities performed during follow-up, Bathia et al. [49], Licker et al. [51], and Karenovics et al. [54] exposed patients to high-intensity interval training. In their study, Li et al. [46], reported that of the 169 patients enrolled in the clinical trial, 86 underwent a program consisting of three essential components: respiratory training, walking, and individualized nutritional support. Wang et al. [48] focused their intervention on respiratory training, developing activities such as abdominal respiratory training, pursed-lip breathing, and incentive spirometry exercises. It is important to note that one of the common elements in most studies is education about the disease and its implications, as well as the importance of exercise as an essential part of the intervention.

Regarding the studies that evaluated the effects of the intervention after the surgical procedure [56,57,58,59,60,61,62,63,64,65,66,67,68,69], similar to what was described earlier, the setting in which the intervention was most frequently implemented was a medical center (71%), followed by studies where the intervention was conducted in two settings (medical center and home) (14.5%), and with a similar percentage, those in which the intervention was performed primarily in the home setting (14.5%). Concerning the number of sessions conducted during follow-up, it is important to note that they were usually higher than those conducted in studies where the intervention was performed before surgery, ranging from 16 to 60 sessions [56,57,58,59,62,63,64,66,68,69]. Regarding this, Yu et al. [56], Ferreira et al. [58], Messaggi et al. [63], and Stigt et al. [68] reported an average of 24 exercise sessions in their studies. The studies with the highest number of sessions were conducted by Edvardsen et al. [66] with 60 sessions, Xu et al. [57] with 48 sessions, and Liu et al. [62] and Rutkowska et al. [64] who similarly reported 30 exercise sessions. Four studies did not precisely report the number of training sessions [60,61,65,67]. Additionally, in 10 of the 14 studies, the intervention was based on aerobic and resistance training [58,59,61,62,63,64,66,67,68,69]. Moreover, in the studies by Liu et al. [62], Messaggi et al. [63], Brocki et al. [65], and Edvardsen et al. [66], inspiratory muscle training was considered an essential element of the intervention. It is important to highlight that, although it was not the primary focus of the study, the exercise prescription was accompanied by other complementary activities such as nutritional and psychological support and education about the disease and the benefits of rehabilitation [57,58,59,60]. 

The details of the exercise-based intervention after surgery are described in Table 4.

### 3.4. Risk of Bias Assessment Report

**Figure 2:**
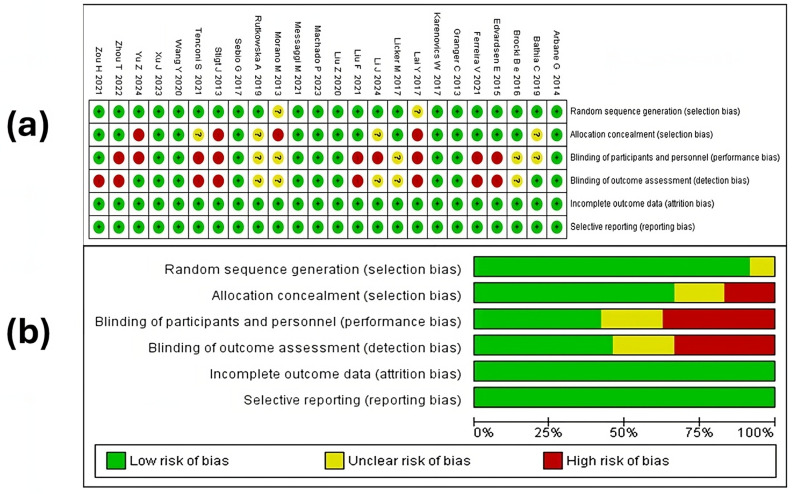
Risk of bias assessment for studies included in this systematic review. (**a**) Risk of bias for each study included in the review, according to the authors’ assessment. The symbol «+» represents a low risk of bias, «?» indicates an unclear risk of bias, and «-» suggests a high risk of bias. The colors used with each symbol are green for low risk, yellow for unclear risk, and red for high risk. (**b**) Summary of the risk of bias as evaluated for each study, with each bias item presented as a percentage [46,47,48,49,50,51,52,53,54,55,56,57,58,59,60,61,62,63,64,65,66,67,68,69].

The risk of bias in the studies was evaluated using various criteria, as illustrated in Figure 2. Below are the key findings from the assessment, as represented in the graph created in RevMan version 5.4^®^ (accessed on 15–23 May 2024).

#### 3.4.1. Random Sequence Generation

The majority of the included studies—Arbane et al., 2014; Bhatia et al., 2019; Brocki et al., 2016; Edvardsen et al., 2015; Ferreira et al., 2021; Granger et al., 2013; Karenovics et al., 2017; Licker et al., 2017; Li J et al., 2024; Li F et al., 2021; Liu et al., 2020; Machado et al., 2023; Messaggi et al., 2021; Rutkowska et al., 2019; Sebio et al., 2017; Stigt et al., 2013; Tenconi et al., 2021; Wang et al., 2020; Xu et al., 2023; Yu et al., 2024; Zhou et al., 2022; Zou et al., 2021—were rated as having a low risk of bias related to random sequence generation (selection bias), indicating that the authors employed a thorough randomization procedure and properly documented it in their publications [46,47,48,49,51,53,54,55,56,57,58,59,60,61,62,63,64,65,66,67,68,69]. However, the studies by Lai Y et al. [50] and Morano et al. [52] were rated as having an “unclear” risk of bias.

#### 3.4.2. Allocation Concealment

Allocation concealment (selection bias) was rated as low risk in 16 of the studies included in the systematic review [47,48,51,53,54,57,58,59,60,61,62,63,65,66,67,69]. However, the studies by Bhatia et al., 2019 [49]; Li et al., 2024 [46]; Rutkowska et al., 2019 [64]; and Tenconi et al., 2021 [55] were rated as having an unclear risk, while those by Lai et al., 2017 [50]; Morano et al., 2013 [52]; Stigt et al., 2013 [68]; and Yu et al. [56] were classified as having a high risk of bias. This suggests the need for study protocols to incorporate this good practice and provide more explicit information on the procedure in scientific publications.

#### 3.4.3. Blinding of Participants and Personnel

Less than half of the studies included in the review presented a low risk of bias related to blinding of participants and personnel [47,48,53,54,57,60,61,63,67,69]. Conversely, 58% of the studies were classified as having an unclear or high risk of bias. Specifically, the studies by Bhatia et al., 2019; Brocki et al., 2016; Licker et al., 2017; Morano et al., 2013; and Rutkowska et al., 2019 were rated as having unclear risk [49,51,52,64,65], while Edvardsen et al., 2015; Ferreira et al., 2021; Lai et al., 2017; Li et al., 2024; Liu et al., 2021; Stigt et al., 2013; Tenconi et al., 2021; Yu et al., 2024; and Zhou et al., 2021 were rated as having a high risk of bias [46,50,55,56,58,59,62,66,68]. This high risk is largely due to the nature of the intervention, which can make blinding challenging. Nevertheless, the lack of blinding can lead to significant issues, as knowing the type of training the patients are receiving might influence their perceptions or those of their evaluators, potentially affecting the results. This underscores the importance of designing RCTs that, where possible, aim to minimize performance bias through various alternatives, even when complete blinding is difficult to implement due to the nature of the intervention. It is also crucial that researchers transparently report the alternatives they adopt and the possible implications of performance bias in their findings.

#### 3.4.4. Blinding of Outcome Assessment

Regarding detection bias, measured through the blinding of outcome assessors, the studies by Edvardsen et al., 2015; Ferreira et al., 2021; Lai et al., 2017; Liu F et al., 2021; Stigt et al., 2013; Tenconi et al., 2021; Zhou et al., 2022; and Zou et al., 2021 were rated as having a high risk of bias [50,55,58,59,60,62,66,68]. Additionally, the studies by Brocki et al., 2016; Licker et al., 2017; Li et al., 2024; Morano et al., 2013; and Rutkowska et al., 2019 were rated as having unclear risk [46,51,52,64,65]. It is important to consider that the lack of blinding among assessors can, in some cases, lead to less objective evaluations of clinical outcomes.

#### 3.4.5. Incomplete Outcomes and Selective Reporting

Concerning the domain of “incomplete outcomes”, all studies were rated as low risk. This indicates that the authors were transparent in reporting participant attrition when applicable, as well as how they managed missing data. Regarding “selective reporting” (reporting bias), as with the previous domain, all studies were categorized as having a low risk of bias. This suggests that the studies included in the review consistently reported all outcomes of interest without omitting those that might be less favorable or non-significant.

Overall, the studies demonstrated a predominantly low risk of bias across most domains, indicating adequate methodological quality in most cases. However, variability in blinding of patients, personnel, and outcome assessors in some reports calls for a cautious interpretation of the findings.

### 3.5. Qualitative Synthesis of Study Results

#### 3.5.1. Health-Related Quality of Life

Health-related quality of life (HRQoL) in patients who received the intervention before surgery was evaluated using the FACT-L, QLQ-C30, SF-12, and SF-36 instruments. In three of the five studies, it was found that HRQoL improved when participants were subjected to an exercise or pulmonary rehabilitation program compared to standard treatment [46,47,53]. In the study by Li et al. [46], after evaluating 169 patients, of whom 86 received the intervention, it was found that at 12 weeks of follow-up, the scores in terms of physical, emotional, functional, and social/family well-being were significantly higher in those who underwent the intervention (*p* < 0.05). Similarly, Machado et al. [47] reported that while 71.4% of the control group experienced a deterioration in HRQoL, only 30% of the rehabilitation group did (*p* = 0.003); significant differences were observed in pain, appetite, physical and emotional function, and role functioning after surgery. Furthermore, Sebio et al. [53] concluded that preoperative rehabilitation not only improves muscle function, physical capacity, and exercise ability but also enhances HRQoL.

These findings contrast with those reported by authors such as Lai et al. [50], who did not identify differences between groups in the global HRQoL score or in any of the dimensions evaluated: physical function, emotional function, and dyspnea related to daily life activities.

On the other hand, a total of seven studies evaluated HRQoL as an outcome in patients who underwent an exercise or pulmonary rehabilitation program after surgery, with the most frequently used questionnaires for this evaluation being the SF-36 and QLQ-C30 [56,58,63,66,67,68,69]. Three of the seven studies suggest that the intervention improves HRQoL [58,66,69]. Ferreira et al. [58] reported that patients in the intervention group had significantly higher HRQoL scores compared to the control group at four weeks of follow-up. The dimensions that showed clear differences between the groups were general health, mental health, physical function, and social function. Additionally, Edvardsen et al. [66] included 61 patients in their study, of whom 30 were assigned to the intervention group. After 20 weeks of follow-up from the start of the program, it was found that there was an improvement in favor of the intervention group in the physical component score (51.8 ± 5.5 vs. 43.3 ± 11.3; *p* = 0.0006), as well as in the mental component score (55.5 ± 5.3 vs. 46.6 ± 14.0; *p* = 0.002) of the SF-36 scale; a reduction in dyspnea scores was also reported in the exercise group compared to the control group (*p* = 0.03). Similarly, Granger et al. [69] found that rehabilitation was associated with positive trends in some domains of HRQoL; for example, in the mental and physical function domains, there was a deterioration in scores in the control group compared to a clear improvement in the intervention group. Moreover, the vitality and general health domains decreased in the control group while remaining relatively stable in the rehabilitation group.

In contrast, four studies [56,63,67,68] agree that HRQoL does not change significantly after enrolling lung cancer patients in pulmonary rehabilitation or exercise programs. In the study by Yu et al. [56], although the QLQ-C30 score was lower in the intervention group, the difference was not clinically or statistically significant (58.4 ± 9.3 vs. 61.7 ± 5.7; *p* = 0.318). Similarly, Messaggi et al. [63] found that while HRQoL scores improved in both groups, no significant differences were observed between them.

#### 3.5.2. Symptom Improvement

A total of six studies evaluated symptom improvement in lung cancer patients undergoing exercise or pulmonary rehabilitation (three studies with pre-surgical intervention and three with post-surgical intervention) [47,48,55,57,60,64]. The symptom evaluated in all studies was the level of dyspnea, measured using the Borg and mMRC scales. Three of the six publications suggest that rehabilitation before or after surgery improves the sensation of dyspnea [48,55,60]. Wang et al., in a population of 65 patients (31 in the intervention group), found that although dyspnea was initially higher in the intervention group than in the control group, respiratory exercises compared to standard care significantly reduced the level of dyspnea after surgery. Additionally, the intervention group showed significantly greater improvement than the control group when analyzing patients with non-small-cell lung cancer and chronic obstructive pulmonary disease (*p* = 0.006). Similarly, Rutkowska et al. [64], although they did not find clinically significant differences in Borg scores within the intervention and control groups at six weeks of follow-up, did identify significant differences when comparing pulmonary rehabilitation groups to standard care (*p* = 0.04).

In contrast to the studies described, the results of Machado et al. [47], Tenconi et al. [55], and Xu et al. [57] suggest that exercise or rehabilitation does not influence the level of dyspnea in lung cancer patients. Xu et al. [57] reported that although dyspnea scores improved significantly within both the intervention and control groups during follow-up, no statistically significant differences were observed between the groups (*p* > 0.05).

#### 3.5.3. Adverse Events

Four authors evaluated [47,50,55,69] adverse events associated with exercise or rehabilitation intervention in lung cancer patients, with all including this outcome as part of the secondary results. Machado et al. [47] defined adverse events for measurement purposes as any unfavorable or unexpected event related to physical training during or within 24 h after a training session. After evaluating 20 patients who received an average of 3.6 ± 0.2 weeks of 17 sessions consisting of aerobic exercises, resistance training, and telephone supervision, they found that six participants (30%) reported mild adverse events, particularly leg muscle pain (20%). Additionally, no serious adverse events related to the intervention were observed. Similarly, in the study by Lai et al. [50], few patients experienced adverse events. According to the authors, during follow-up, four participants had to discontinue training because they could not tolerate the high-intensity regimen, one withdrew due to a perceived lack of benefits, and one experienced knee pain. Tenconi et al. [55] reported a total of 84 adverse events during the follow-up period, of which 30 occurred in the intervention group. Of all documented cases, 52.4% were related to the intervention, and in 21.4%, the possible correlation could not be determined. Eleven adverse events in the rehabilitation arm required hospitalization, and one was considered potentially life-threatening. In contrast to these findings, Granger et al. [69] did not document adverse events during stress tests, exercise training for hospitalized patients, or exercise training in the home setting.

#### 3.5.4. Mortality

A total of three studies evaluated mortality in lung cancer patients who received exercise or pulmonary rehabilitation intervention before or after surgery [51,58,61]. Liu et al. [61] conducted a study on 73 patients, of whom 37 participated in an intervention consisting of aerobic and resistance exercises, respiratory training, nutritional counseling, and psychological support. After follow-up, they found that none of the participants in either group died. On the other hand, Licker et al. [51] evaluated whether a preoperative high-intensity interval training program improves cardiorespiratory capacity and reduces postoperative complications in lung cancer patients. When comparing mortality in 77 patients who received standard follow-up versus 74 enrolled in a pulmonary rehabilitation program, they found that 30-day mortality was low, and there were no clinically or statistically significant differences between groups (intervention 2.7% vs. control 2.6%; *p* = 0.640). Similarly, Ferreira et al. [58] reported that few patients died following an exercise intervention (2/52 people).

### 3.6. Meta-Analysis Results

A meta-analysis was conducted for three outcomes based on the studies published by Karenovics et al. [54], Lai et al. [50], Li et al. [46], Morano et al. [52], Xu et al. [57], Yu et al. [56], Zhu et al. [59], Zou et al. [60], Rutkowska et al. [64], Bhatia et al. [49], Tenconi et al. [55], Granger et al. [69], Liu et al. [61], and Licker et al. [51]. The results are presented separately based on the timing of the intervention (pre-surgical or post-surgical).

#### 3.6.1. Assessment of the Quality of the Evidence

Among the 14 studies evaluated, 12 received scores of 4 points or higher, indicating that most trials met the criteria for randomization, proper description of withdrawals, appropriate execution of randomization procedures, and clear presentation of inclusion and exclusion criteria. However, the implementation of a double-blind design was limited; only one study [69] earned a point in this category, achieving the maximum possible score of 5 points. The absence of blinding in some studies may be attributed to the nature of the intervention—pulmonary rehabilitation—which can complicate the implementation of blinding procedures. Refer to Table 5 for details.

#### 3.6.2. Lung Function

**Figure 3:**
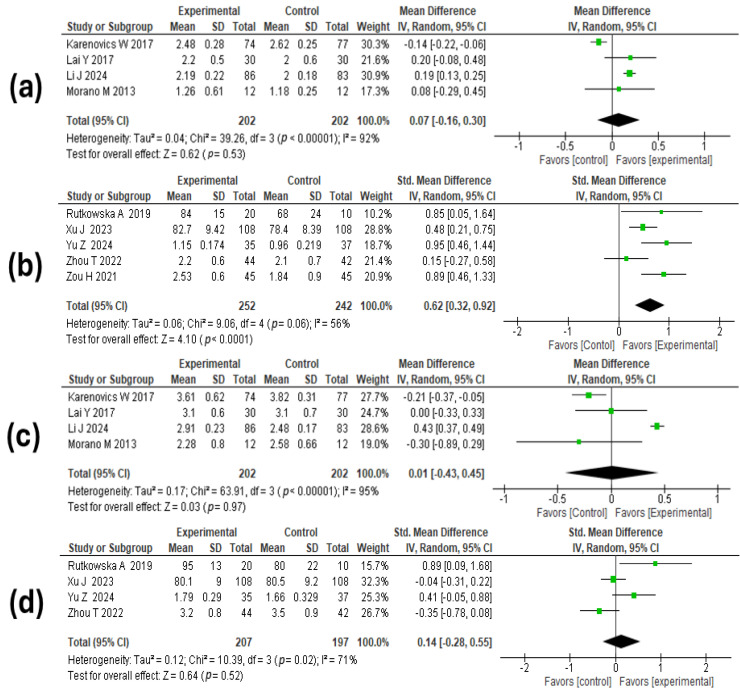
Forest plots estimating the effect of exercise-based rehabilitation on lung function. (**a**) Effect of pre-surgical exercise-based rehabilitation vs. control on FEV1 values. (**b**) Effect of post-surgical exercise-based rehabilitation vs. control on FEV1 values. (**c**) Effect of pre-surgical exercise-based rehabilitation vs. control on FVC values. (**d**) Effect of post-surgical exercise-based rehabilitation vs. control on FVC values [46,50,51,52,56,57,59,60,64].

A total of nine studies [46,50,51,52,56,57,59,60,64], including 898 participants (404 pre-surgical and 494 post-surgical), evaluated this outcome. For the evaluation of FEV1 in patients who received the intervention before surgery, a χ^2^ = 39.2, *p* < 0.00001, I^2^ = 92% was found. The meta-analysis results showed that the mean difference in FEV1 between the exercise or rehabilitation group and the standard care group was 0.07 (95% CI: −0.16 to 0.30; *p* = 0.53). Regarding FEV1 in participants who received exercise or rehabilitation training after surgery, a χ^2^ = 39.06, *p* = 0.06, I^2^ = 56% was observed. The meta-analysis results showed that patients in the intervention group had higher forced expiratory volume in one second compared to the control arm: standardized mean difference 0.62 (95% CI: 0.32 to 0.92; *p* = 0.0001). See Figure 3a,b.

On the other hand, the meta-analysis shows that, regardless of the timing of the intervention, forced vital capacity in the rehabilitation group did not show statistically significant differences compared to the standard care group. The mean difference in the comparison of rehabilitation versus standard care pre-surgery was 0.01 (95% CI: −0.43 to 0.45; *p* = 0.97); the standardized mean difference in the comparison of rehabilitation versus standard care post-surgery: 0.14 (95% CI: −0.28 to 0.55; *p* = 0.52). See Figure 3c,d.

#### 3.6.3. Walking Distance

Twelve studies, including a total of 1050 patients (487 pre-surgical and 560 post-surgical), evaluated this outcome. Regarding the 6 min walking distance among patients who received the intervention before surgery compared to standard care, a χ^2^ = 9.97, *p* < 0.004, I^2^ = 60% was found. The meta-analysis results showed that the mean difference in the 6 min walking distance between the exercise or rehabilitation group and the standard care group was 37.42 (95% CI: 9.68 to 65.1; *p* = 0.008). This suggests that the walking distance is significantly higher in patients prescribed exercise or pulmonary rehabilitation compared to those receiving conventional care.

**Figure 4:**
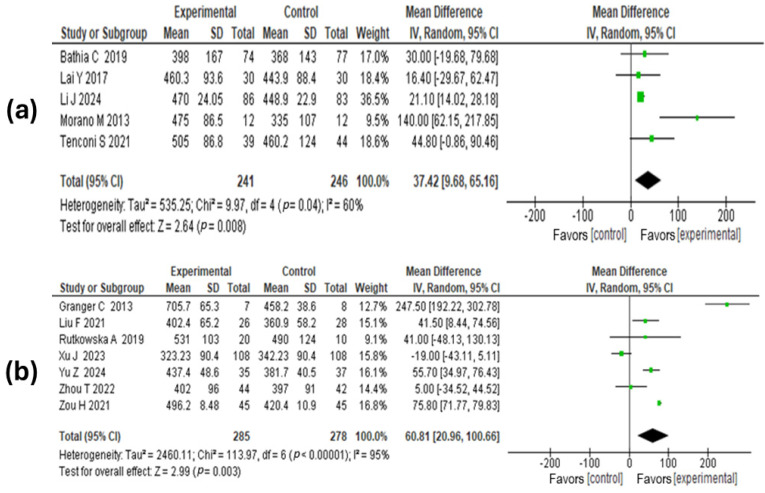
Forest plots estimating the effect of exercise-based rehabilitation on the 6-min walking distance. (**a**) Effect of pre-surgical exercise-based rehabilitation vs. control on 6 min walking distance. (**b**) Effect of post-surgical exercise-based rehabilitation vs. control on 6 min walking distance [46,49,50,52,55,56,57,60,62,64,69].

Regarding the 6 min walking distance among subjects who underwent the intervention after surgery, a χ^2^ = 113.9, *p* = 0.0001, I^2^ = 95% was observed. The meta-analysis results suggest that patients in the intervention group walked greater distances in 6 min compared to the control arm: standardized mean difference 60.8 (95% CI: 20.96 to 100.6, *p* = 0.0033). See Figure 4.

#### 3.6.4. Hospital Stay

**Figure 5:**
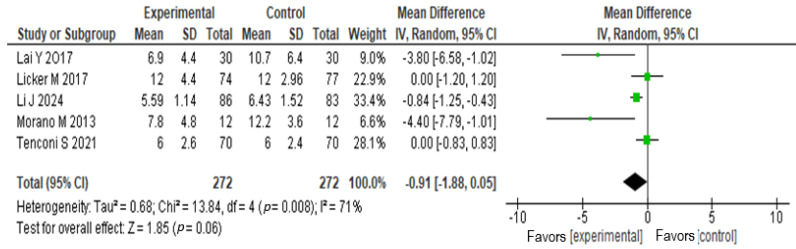
Forest plots estimating the effect of pre-surgical exercise-based rehabilitation on hospital stay [46,50,51,52,55].

Five studies [46,50,51,52,55] evaluated the hospital stay in patients who received exercise or pulmonary rehabilitation intervention before surgery. A χ^2^ = 13.8, *p* < 0.0008, I^2^ = 71% was found. The meta-analysis results showed that the mean difference in hospital stay between the exercise or rehabilitation group and the standard care group was −0.91 (95% CI: −1.88 to 0.055; *p* = 0.06). These findings suggest that there is no statistically significant difference between the two groups. See Figure 5.

## 4. Discussion

The randomized controlled trials included in this systematic review reveal significant findings regarding the effects of rehabilitation and exercise in patients undergoing surgery for lung cancer. A total of 24 studies were included, of which 10 evaluated pre-surgical interventions [46,47,48,49,50,51,52,53,54,55] and 14 post-surgical interventions [56,57,58,59,60,61,62,63,64,65,66,67,68,69], providing a comprehensive overview of the different phases of treatment for patients diagnosed with lung cancer.

Most of the studies were published recently, with 88% conducted in Europe and Asia (50% and 38%, respectively) [46,47,48,49,50,51,53,54,55,56,57,59,60,61,62,63,64,65,66,67,68,69]. This finding suggests a growing trend in developing studies on exercise and rehabilitation as an integral part of lung cancer patient management. The frequency of publications on this topic in the mentioned continents may be related to the robustness of their healthcare systems, which generally translates into more resources allocated for oncology patient follow-up.

In the pre-surgical studies, 70% of the interventions were carried out in healthcare centers, while in 20% of the studies, rehabilitation or exercise was conducted in a combination of medical centers and homes. This pattern is similar to that observed in the post-surgical studies (healthcare center: 71%, home: 14.5%, and both settings: 14.5%). This finding indicates that regardless of when the intervention is conducted, there is a preference for healthcare centers as the primary setting for rehabilitation. This preference is due to the need for professional supervision and a controlled environment to monitor the patient’s response and adjust the interventions when necessary. Additionally, some interventions described in the studies require the use of specific devices or technologies, such as specialized exercise equipment, functional capacity measurement tools, and monitoring systems, which are primarily available in clinical settings [64,68,70].

On the other hand, using the home environment for exercise and rehabilitation can facilitate treatment adherence and provide additional comfort for patients, allowing them to perform activities in a familiar and accessible setting. This alternative can benefit patients with difficulties traveling to a healthcare center, reducing logistical and emotional barriers. However, it is essential to highlight that the home setting poses some challenges:

- The lack of direct supervision and specialized resources at home can limit the effectiveness of interventions.
- The absence of a controlled and supervised environment may increase the risk of errors in technique, as patients might not receive the necessary guidance to perform exercises correctly.
- The lack of supervision could lead to a reduction in the overall efficacy of the intervention, potentially resulting in suboptimal outcomes [71,72,73,74].

Despite these challenges, some studies have demonstrated the safety of exercise or rehabilitation at home. For instance, Coats et al. [75] reported that a 4-week home-based rehabilitation program for pre-surgical lung cancer patients was safe and feasible. Similarly, Rispoli et al. [76], in a study of 59 patients with a history of COPD undergoing preoperative home rehabilitation, found that the intervention was associated with improved lung function and distance walked, suggesting significant clinical benefits despite the limitations of the home environment. Additionally, Edbrooke et al. [77] concluded in their research that home rehabilitation was acceptable to most participants and reported multiple benefits, including increased motivation, better physical condition, and improved symptom control.

The duration and number of sessions of pre- and post-surgical interventions varied considerably among the authors, ranging from one to twenty weeks. In the pre-surgical context, Li et al. [46] and Bhatia et al. [49] implemented two- to three-week programs, while Lai et al. [50] conducted a one-week program, and Morano et al. [52] opted for a more extended four-week program. When the intervention was pre-surgical, there was also considerable variation, although the durations were generally longer. Xu et al. [57] implemented a 12-week program, while Ferreira et al. [58] and Zou et al. [60] used 8-week programs. Liu et al. [62] and Rutkowska et al. [64] conducted 6-week programs.

The variability in the duration of these programs suggests that shorter interventions can be effective in some cases, but longer programs may provide greater benefits in other outcomes. Spruit et al. [78] noted that interventions designed to improve cardiorespiratory fitness in chronic respiratory conditions should typically last around 8 to 12 weeks, as longer periods yield better outcomes. However, it is important to consider that the stage of lung cancer sometimes necessitates immediate surgical interventions, limiting the time available for other management options, such as exercise and rehabilitation.

HRQoL is a crucial aspect of managing lung cancer patients undergoing surgery. Therefore, the goal of rehabilitation or exercise programs before or after surgery is to improve this dimension [79,80]. Despite this, the studies in this review show considerable variability in the impact of these interventions. In general, several studies demonstrated significant improvements in HRQoL when patients participated in rehabilitation or exercise programs before surgery, showing better scores in the physical, emotional, and social dimensions. However, this trend is not universal, as some studies did not find significant differences in HRQoL between patients who received the intervention and those who followed standard treatment [53,55,56,63,66,67,68,69].

The discrepancies highlighted above are reflected in previously published studies with different designs from RCTs. Some authors argue that rehabilitation or exercise may result in little or no change in HRQoL [69,81,82]. In contrast, a systematic review by Gravier et al. [83] revealed that pre-surgical exercise training improved patients’ exercise capacity, significantly enhanced lung function, and notably improved HRQoL, also impacting depression levels.

The divergences observed may be explained by several factors. Firstly, the lack of standardization in programs can lead to significant differences in the outcomes obtained. Intensive and longer programs may provide more benefits compared to those without these characteristics. Secondly, the sociodemographic and clinical characteristics of the subjects included in the studies may influence the results. Variables such as age, general health status at the beginning of the intervention, comorbidities, and cancer stage can affect the response to treatment [84,85]. These factors can also alter the perception of HRQoL, particularly if the benefits are not clearly visible. Additionally, variability in the tools used to measure HRQoL is another possible reason for differences between studies. Different questionnaires and scales (FACT-L, QLQ-C30, SF-12, and SF-36) may have varying sensitivities and specificities for detecting changes in HRQoL or may emphasize different dimensions, which could explain why some studies find significant improvements while others do not [86,87,88]. 

On the other hand, the reviewed studies suggest that while exercise and pulmonary rehabilitation interventions are safe and do not increase postoperative mortality in lung cancer patients, they do not seem to significantly reduce the risk of death on their own. Postoperative mortality in these patients may be influenced by multiple factors, and some of these factors may have a stronger impact on the outcomes [89,90]. These results are consistent with other authors’ reports; for example, in their systematic review, Mao et al. [36] reported a pooled OR of 1.32 (95% CI: 0.54–3.23) for the pulmonary rehabilitation group, suggesting that this intervention does not appear to have implications for this outcome. Despite this, they concluded that while pulmonary rehabilitation may not affect mortality in patients undergoing lung resection, it offers other benefits, such as reducing the number of postoperative complications, particularly pulmonary complications. Similarly, Chen et al. [91], in a review of nine prospective clinical studies involving a total of 1338 patients, reported no significant difference in postoperative mortality between patients undergoing rehabilitation compared to standard care (OR = 0.77; 95% CI: 0.26–2.30; *p* = 0.65).

In our meta-analysis, no significant improvements in FEV1 were observed when the intervention was pre-surgical compared to standard care. However, the post-surgical intervention showed a significant improvement in FEV1. Additionally, FVC did not show statistically significant differences compared to standard care, both pre-surgically and post-surgically. The results of preliminary studies on this topic are heterogeneous; for example, in their meta-analysis, García et al. [92] found that both FVC and FEV1 improved significantly after pre-surgical intervention (SMD = 0.38; 95% CI: 0.14–0.63 and SMD = 0.27; 95% CI: 0.11–0.42, respectively). It is important to note that some variables may explain these discrepancies, such as the type of intervention, the timing of the intervention, the patient’s baseline condition, and adherence to the program [93].

Regarding the distance walked in 6 min, this meta-analysis showed that it was consistently higher in patients who exercised or participated in pulmonary rehabilitation before or after surgery compared to standard treatment. This finding is consistent with a previous review, which reported a mean difference of 18.23 m in favor of rehabilitation or exercise [94]. On the other hand, it is worth mentioning that while the impact of the intervention on hospital stay was not clear in this study, preliminary studies have documented shorter hospital stays when patients engage in exercise or pulmonary rehabilitation [53].

It is important to clarify that although this review did not demonstrate the impact of risk factors on the outcomes, there are studies indicating that factors such as smoking, the presence of comorbidities like chronic obstructive pulmonary disease (COPD), baseline functional performance, or the stage of cancer at the time of diagnosis can have a significant impact on the results of rehabilitation and exercise interventions in patients with lung cancer [95,96,97,98]. These variables can not only affect the response to treatment but also influence postoperative recovery and the patient’s quality of life. The omission of these factors in our analysis constitutes a limitation that should be addressed in future reviews. However, it is necessary to consider that in randomized clinical trials, these factors are usually controlled or evenly distributed between the intervention and control groups, which reduces their impact on effectiveness analyses. Despite this, it is crucial to consider that in real clinical settings, where these factors are not always balanced, they can influence the effectiveness of interventions.

Therefore, a more detailed evaluation of risk factors, including not only their presence but also their severity, would allow for a better understanding of rehabilitation outcomes in this patient group. Additionally, it would provide valuable information to personalize rehabilitation interventions according to the individual needs of patients, which could maximize benefits and improve the quality of life for those facing lung cancer.

### 4.1. Limitations

Firstly, it is important to mention that the heterogeneity of the studies in terms of outcome reporting and design limits the ability to make definitive recommendations. Future studies should aim to standardize interventions and outcomes evaluated, which would allow for a better understanding and clearer synthesis of the evidence. Additionally, it is crucial to improve blinding in studies and clearly document randomization to ensure the generalizability of the results. There was significant variability in the duration and intensity of the exercise interventions, making it difficult to determine the optimal intervention time to maximize benefits for lung cancer patients. On the other hand, although this review included a total of 24 studies, it is important to note that the outcomes reported in the meta-analysis are based on a smaller number of investigations, some with considerable heterogeneity and small sample sizes [46,49,50,51,52,54,55,56,57,60,61,64,69]. This variability among the studies may have influenced the consistency of the results, which limits the generalization of the findings and necessitates cautious interpretation. Consequently, there is a justified need for additional, larger-scale clinical trials with more homogeneous methodologies.

### 4.2. Challenges in Applying the Findings

It is important to consider that local conditions in some countries, such as available resources, healthcare infrastructure, and established clinical practices, can vary significantly from those in the settings where the studies included in this review were conducted. This variability may limit the applicability of the recommended interventions in some regions with limited resources or different healthcare systems. For example, in countries with less infrastructure for rehabilitation or significant economic barriers, implementing programs based on studies conducted in developed countries may not be feasible. This challenge highlights the need to adapt and contextualize rehabilitation strategies to be relevant and effective in a wider range of settings.

## 5. Conclusions

This review suggests that, depending on the timing of the intervention, pulmonary rehabilitation or exercise could have positive effects on certain clinical variables in patients with lung cancer. However, the impact of rehabilitation on outcomes such as health-related quality of life, mortality, and hospital stay remains controversial. Future studies should consider standardizing interventions, including factors such as the duration of follow-up for patients with lung cancer.

## Figures and Tables

**Table 1:** Summary of Clinical Trials Included in the Systematic Review with Pre-Surgical Intervention.

Author, Year	Country	Disease	Patients I/C	Sex (% Male)	Age (Years)	Time of Enrollment	Program Duration	Evaluated Outcomes
Li J et al., 2024 [46]	China	NSCLC	I: 86 C: 83	40%	57.7	Pre-surgical	2 weeks	Health-related quality of life, 6 min walking distance, hospital stay, and lung function.
Machado P et al., 2023 [47]	Portugal	NSCLC	I: 20 C: 21	68.30%	68.1	Pre-surgical	NS	Health-related quality of life, 6 min walking distance, dyspnea improvement, and adverse events.
Wang Y et al., 2020 [48]	China	NSCLC, SCLC	I: 31 C: 34	34%	57.2	Pre-surgical	NS	6 min walking distance and dyspnea improvement.
Bhatia C et al., 2019 [49]	Switzerland	NSCLC	I: 74 C: 77	60%	64	Pre-surgical	2–3 weeks	Lung function and 6 min walking distance.
Lai Y et al., 2017 [50]	China	NSCLC	I: 30 C: 30	57%	72	Pre-surgical	1 week	Lung function, quality of life, 6 min walking distance, adverse events, and hospital stay.
Licker M et al., 2017 [51]	Switzerland	NSCLC	I: 74 C: 77	60%	64	Pre-surgical	NS	6 min walking distance, mortality, and hospital stay.
Morano M et al., 2013 [52]	Brazil	NSCLC	I: 12 C: 12	37.50%	66.4	Pre-surgical	4 weeks	Lung function, 6 min walking distance, and hospital stay.
Sebio G et al., 2017 [53]	Spain	NSCLC	I: 10 C: 12	90%	70	Pre-surgical	NS	Health-related quality of life.
Karenovics W et al., 2017 [54]	Switzerland	NSCLC	I: 74 C: 77	60%	64	Pre-surgical	NS	Lung function and health-related quality of life.
Tenconi S et al., 2021 [55]	Italy	NSCLC	I: 70 C: 70	61.40%	66.8	Pre-surgical	NS	6 min walking distance, dyspnea improvement, adverse events, and hospital stay.

NS: Not Specified.

**Table 2:** Summary of Clinical Trials Included in the Systematic Review with Post-Surgical Intervention.

Author, Year	Country	Disease	Patients I/C	Sex (% Male)	Age (Years)	Time of Enrollment	Program Duration	Evaluated Outcomes
Yu Z et al., 2024 [56]	China	NSCLC, COPD	I: 44 C: 40	68%	69	Post-surgical	2 weeks	Lung function, health-related quality of life, and 6 min walking distance.
Xu J et al., 2023 [57]	China	NSCLC	I: 108 C:108	NS	NS	Post-surgical	12 weeks	Lung function, 6 min walking distance, and dyspnea improvement.
Ferreira V et al., 2021 [58]	Canada	NSCLC	I: 43 C: 52	53.60%	66.9	Post-surgical	8 weeks	Health-related quality of life, 6 min walking distance, and hospital stay.
Zhou T et al., 2022 [59]	China	NSCLC	I: 44 C: 42	57%	61.7	Post-surgical	2 weeks	Lung function, 6 min walking distance, and hospital stay.
Zou H et al., 2021 [60]	China	NSCLC	I: 45 C: 45	49%	58.4	Post-surgical	8 weeks	Lung function, 6 min walking distance, dyspnea improvement, and hospital stay.
Liu Z et al., 2020 [61]	China	NSCLC	I: 37 C: 36	31.50%	56.2	Post-surgical	2 weeks	Lung function, 6 min walking distance, mortality, and hospital stay.
Liu F et al., 2021 [62]	Taiwan	NSCLC, SCLC	I: 32 C: 31	41%	65.2	Post-surgical	6 weeks	6 min walking distance.
Messaggi M et al., 2019 [63]	Spain	NSCLC	I: 16 C:21	70.30%	64.6	Post-surgical	8 weeks	Health-related quality of life.
Rutkowska A et al., 2019 [64].	Poland	NSCLC	I: 20 C: 10	90%	60.2	Post-surgical	6 weeks	Lung function, 6 min walking distance, and dyspnea improvement.
Brocki B et al., 2016 [65]	Denmark	NSCLC, SCLC	I: 34 C: 30	57.50%	70	Post-surgical	2 weeks	Lung function and 6 min walking distance.
Edvardsen E et al., 2015 [66]	Norway	NSCLC	I: 30 C: 31	46%	65.1	Post-surgical	20 weeks	Health-related quality of life.
Arbane G et al., 2014 [67]	United Kingdom	NSCLC	I: 64 C:67	55%	68	Post-surgical	NS	Health-related quality of life.
Stigt JA et al., 2013 [68]	Netherlands	NSCLC	I: 23 C: 26	82%	63.4	Post-surgical	12 weeks	Lung function, health-related quality of life, and 6 min walking distance.
Granger C et al., 2013 [69]	Australia	NSCLC, SCLC	I: 7 C: 8	53%	65.5	Post-surgical	8 weeks	Health-related quality of life, 6 min walking distance, and adverse events.

NS: Not Specified.

**Table 3:** Characteristics of the Pre-Surgical Intervention.

Author, Year	Primary Setting	Activities	Number of Sessions	Results
Li J et al., 2024 [46]	Health center	Respiratory training, walking, and individualized nutritional support.	NS	PR combined with nutritional support promotes better recovery and higher quality of life after surgery.
Machado P et al., 2023 [47]	Home	Educational session, aerobic exercises, resistance training, and telephone supervision.	17	PR can effectively prevent the decline in quality of life after lung cancer surgery.
Wang Y et al., 2020 [48]	Health center	Abdominal respiratory training, pursed-lip breathing, and incentive spirometry exercises.	NS	Pre-surgical respiratory exercise can relieve dyspnea, improve inspiratory capacity, and reduce dyspnea level.
Bhatia C et al., 2019 [49]	Health center	High-intensity interval training.	8	High-intensity training is safe in the preoperative period and increases cardiorespiratory fitness.
Lai Y et al., 2017 [50]	Health center	Resistance training and inspiratory muscle training.	7	The program led to a positive effect on peak expiratory flow and 6 min walking distance.
Licker M et al., 2017 [51]	Health center	High-intensity interval training.	NS	The intervention was safe and brought short-term positive effects for patients awaiting lung cancer surgery.
Morano M et al., 2013 [52]	Health center	Upper limb strength training, resistance training, and inspiratory muscle training.	20	Pre-surgical pulmonary rehabilitation improves functional capacity and reduces postoperative respiratory morbidity.
Sebio G et al., 2017 [53]	Health center and home	Resistance training, aerobic training, and respiratory exercises.	NS	A pre-surgical pulmonary rehabilitation program appears to improve patients’ preoperative condition and may prevent functional decline after surgery.
Karenovics W et al., 2017 [54]	Health center	High-intensity interval training.	NS	Short-term high-intensity preoperative rehabilitation does not improve lung function 1 year after lung cancer resection.
Tenconi S et al., 2021 [55]	Health center and home	Aerobic training, resistance training, inspiratory muscle training, and education.	39	Rehabilitation was associated with greater exercise tolerance. No differences were found in quality of life.

NS: Not Specified.

**Table 4:** Characteristics of the Post-Surgical Intervention.

Author, Year	Setting	Activities	Number of Sessions	Results
Yu Z et al., 2024 [56]	Health center	Early mobilization, coughing technique, and aerobic training.	24	A short-term post-surgical exercise training program can facilitate the recovery of functional capacity in lung cancer patients.
Xu J et al., 2023 [57]	Health center and home	Respiratory exercises plus education.	48	The intervention, like conventional care, improved postoperative lung function and the quality of life of the studied patients.
Ferreira V et al., 2021 [58]	Home	Aerobic exercises, resistance training, and psychological and nutritional support.	24	Rehabilitation initiated 4 weeks before surgery is as effective in recovering functional capacity as postoperative rehabilitation.
Zhou T et al., 2022 [59]	Health center	Aerobic training, strengthening of intercostal muscles and abdominal muscles, and education.	14	Rehabilitation could improve early lung function in lung cancer patients after a thoracoscopic lobectomy and reduce the length of hospital stay.
Zou H et al., 2021 [60]	Health center	Respiratory and lower limb training, positive expiratory pressure breathing exercises, cycling, dancing, and education.	NS	Pulmonary rehabilitation could effectively improve lung function, exercise tolerance, and reduce postoperative hospital stay in lung cancer patients.
Liu Z et al., 2020 [61]	Home	Aerobic and resistance exercises, respiratory training, and nutritional and psychological counseling.	NS	The intervention was associated with clinically relevant improvements in perioperative functional capacity in lung cancer patients.
Liu F et al., 2021 [62]	Health center	Inspiratory muscle training and aerobic exercises.	30	Patients who underwent the intervention showed significant improvements starting from two weeks.
Messaggi M et al., 2021 [63]	Health center	Continuous aerobic training and inspiratory and expiratory muscle training.	24	The intervention improved exercise capacity but had no impact on quality of life.
Rutkowska A et al., 2019 [64]	Health center	Respiratory training and aerobic, resistance, and relaxation exercises.	30	The training program was associated with a greater walking distance. Additionally, an improvement in lung function was observed.
Brocki B et al., 2016 [65]	Health center and home	Inspiratory muscle training plus respiratory exercises.	NS	The intervention did not have a significant impact on the evaluated outcomes; however, it improved lung oxygenation.
Edvardsen E et al., 2015 [66]	Health center	High-intensity strength and resistance training. Inspiratory muscle training.	60	The intervention was associated with improved quality of life in lung cancer patients.
Arbane G et al., 2014 [67]	Health center	Daily mobilization, strength training, and aerobic training.	NS	There were no differences in walking distance or quality of life between the intervention and control groups.
Stigt JA et al., 2013 [68]	Health center	Muscle training and aerobic exercises.	24	Rehabilitation did not result in better quality of life in the studied lung cancer patients.
Granger C et al., 2013 [69]	Health center	Aerobic and resistance training.	16	The intervention was associated with positive trends in the 6 min walk test and some domains of quality of life.

NS: Not Specified.

**Table 5:** Assessment of the Quality of Evidence of the Studies Included in the Meta-Analysis with Jadad Score.

Author	The Study Is Randomized	The Intervention Is Double-Blind	Study Withdrawals Are Accounted for and Described	The Randomization Procedure Is Adequate	Selection Criteria	Score
Karenovics et al., 2017 [54]	1	0	1	1	1	4
Lai et al., 2017 [50]	1	0	1	0	1	3
Li et al., 2024 [46]	1	0	1	1	1	4
Morano et al., 2013 [52]	1	0	1	1	1	4
Xu et al., 2023 [57]	1	0	1	1	1	4
Yu et al., 2024 [56]	1	0	1	1	1	4
Zhou et al., 2020 [59]	1	0	1	1	1	4
Zou et al., 2021 [60]	1	0	1	1	1	4
Rutkowska et al., 2019 [64]	1	0	1	1	1	4
Bhatia et al., 2019 [49]	1	0	1	1	1	4
Tenconi et al., 2021 [55]	1	0	1	0	1	3
Granger et al., 2013 [69]	1	1	1	1	1	5
Liu et al., 2020 [61]	1	0	1	1	1	4
Licker et al., 2017 [51]	1	0	1	1	1	4

## Data Availability

Not applicable.

## Abbreviations

NSCLC	Non-Small-Cell Lung Cancer
SCLC	Small Cell Lung Cancer
SF-36	Short Form 36 Health Survey
SF-12	Short Form 12 Health Survey
FACT-L	Functional Assessment of Cancer Therapy—Lung
QLQ-C30	Quality of Life Questionnaire—Core 30
RCT	Randomized Controlled Trial
COPD	Chronic Obstructive Pulmonary Disease
LC	Lung Cancer
PR	Pulmonary Rehabilitation
FEV1	Forced Expiratory Volume in 1 s
FVC	Forced Vital Capacity
